# Supplementary material for: A Critical View of the Application of the APEX Software (Aqueous Photochemistry of Environmentally-Occurring Xenobiotics) to Predict Photoreaction Kinetics in Surface Freshwaters
Source: Molecules. 2019 Dec 18;25(1):9. doi: 10.3390/molecules25010009 (PMC7017383; doi:10.3390/molecules25010009)
Supplement: Supplementary file 1 [file molecules-25-00009-s001.zip › 3.2.4_gcc-4.4.0.zip!/3.2.4_gcc-4.4.0/3.2.4_gcc-4.4.0/doc/octave/html/interpreter/File-Positioning.html]

File Positioning - Untitled


Previous: EOF and Errors,
Up: C-Style I/O Functions


---

#### 14.2.19 File Positioning

Three functions are available for setting and determining the position of
the file pointer for a given file.

— Built-in Function: **ftell** (fid)  
> Return the position of the file pointer as the number of characters
> from the beginning of the file fid.
>
>
>
> **See also:** fseek, fopen, fclose.

— Built-in Function: **fseek** (fid, offset, origin)  
> Set the file pointer to any location within the file fid.
>
> The pointer is positioned offset characters from the origin,
> which may be one of the predefined variables `SEEK_CUR` (current
> position), `SEEK_SET` (beginning), or `SEEK_END` (end of
> file) or strings "cof", "bof" or "eof". If origin is omitted,
> `SEEK_SET` is assumed. The offset must be zero, or a value returned
> by `ftell` (in which case origin must be `SEEK_SET`).
>
> Return 0 on success and -1 on error.
>
>
>
> **See also:** ftell, fopen, fclose.

— Built-in Function: **SEEK\_SET** ()  
— Built-in Function: **SEEK\_CUR** ()  
— Built-in Function: **SEEK\_END** ()  
> Return the value required to request that `fseek` perform
> one of the following actions:
>
> `SEEK_SET`: Position file relative to the beginning. `SEEK_CUR`: Position file relative to the current position. `SEEK_END`: Position file relative to the end.

— Built-in Function: **frewind** (fid)  
> Move the file pointer to the beginning of the file fid, returning
> 0 for success, and -1 if an error was encountered. It is equivalent to
> `fseek (`fid`, 0, SEEK_SET)`.

The following example stores the current file position in the variable
`marker`, moves the pointer to the beginning of the file, reads
four characters, and then returns to the original position.

```
     marker = ftell (myfile);
     frewind (myfile);
     fourch = fgets (myfile, 4);
     fseek (myfile, marker, SEEK_SET);
```
